# Insulin Resistance and Type 2 Diabetes Mellitus: An Ultimatum to Renal Physiology

**DOI:** 10.7759/cureus.28944

**Published:** 2022-09-08

**Authors:** Susmita Sinha, Mainul Haque

**Affiliations:** 1 Physiology, Khulna City Medical College and Hospital, Khulna, BGD; 2 Pharmacology and Therapeutics, National Defence University of Malaysia, Kuala Lumpur, MYS

**Keywords:** end-stage renal disease (esrd), chronic kidney disease, diabetes mellitus, podocytes, renal tubular cells, pi3k pathway, vasodilation, nitric oxide, insulin resistance, insulin

## Abstract

Insulin resistance (IR) is stated as diminished insulin action regardless of hyperinsulinemia. The usual target organs for insulin activities are the liver, skeletal muscle, and adipose tissue. Hence, the vasculature and kidneys are nonconventional target organs as the impacts of insulin on these are comparatively separate from other conventional target organs. Vasodilation is achieved by raising endothelial nitric oxide (NO) generation by initiating the phosphoinositide 3-kinase (PI3K) pathway. In insulin-nonresponsive conditions, this process is defective, and there is increased production of endothelin-1 through the mitogen-activated protein kinase/extracellular signal-regulated kinase (MAPK/ERK) pathway, which predominates the NO effects, causing vasoconstriction. Renal tubular cells and podocytes have insulin receptors, and their purposeful importance has been studied, which discloses critical acts of insulin signaling in podocyte survivability and tubular action. Diabetic nephropathy (DN) is a prevalent problem in individuals with hypertension, poor glycemic management, hereditary susceptibility, or glomerular hyperfiltration. DN could be a significant contributing factor to end-stage renal disease (ESRD) that results from chronic kidney disease (CKD). IR and diabetes mellitus (DM) are the constituents of syndrome X and are accompanied by CKD progression. IR performs a key part in syndrome X leading to CKD. However, it is indistinct whether IR individually participates in enhancing the threat to CKD advancement rather than CKD complexity. CKD is an extensive public health problem affecting millions of individuals worldwide. The tremendous spread of kidney disease intensifies people’s health impacts related to communicable and noncommunicable diseases. Chronic disease regulator policies do not include CKD at global, local, and/or general levels. Improved knowledge of the character of CKD-associated problems might aid in reforming diagnosis, prevention, and management.

## Introduction and background

The incidence and prevalence of end-stage renal disease (ESRD) and chronic kidney disease (CKD) are rising alarmingly around the world [1]. Numerous metabolic abnormalities, including oxidative stress, ongoing inflammation, and endothelial dysfunction, are present in CKD [2]. Insulin resistance (IR) in advanced renal disease is a widely known condition and is one of the elements causing the rise in mortality due to CKD [3]. Due to the high occurrence of diabetic nephropathy (DN) in those with poorly managed diabetes, diabetes mellitus (DM) continues to be a serious health concern. DN is the most typical microvascular consequence of DM that might result in ESRD. DN is characterized by proteinuria in the absence of any other renal disease. This issue frequently affects people with hypertension, poor glycemic control, inherited susceptibility, or glomerular hyperfiltration [4]. IR is crucial for the onset and development of DN. According to a report, people with type 2 diabetes mellitus (T2DM) with DN are more insulin-resistant than those without [5]. One of the primary signs of DN is microalbuminuria, which is directly related to IR [6]. In diabetic kidney disease/DN, various pathological processes are motivated by podocytes’ insulin signal transmission pathway errors.

The mechanisms underlying the establishment of IR in podocytes must be understood to comprehend the morphological and functional degeneration of podocytes, glomeruli, and, ultimately, the kidneys in diabetes [7]. Numerous investigations found a direct link between DN, structural damage, and dysfunction of glomerular podocytes [8]. Proteinuria and glomerulosclerosis are caused by reductions in the number of podocytes equally in diabetic and nondiabetic glomerular disorders [9]. IR and compensatory hyperinsulinemia are linked to a higher prevalence of CKD. Previous research revealed that several processes connect IR and hyperinsulinemia to renal injury [10]. In addition, insulin promotes renal cell growth and encourages the origination of significant growth promoters such as insulin-like growth factor-1 (IGF-1) and transforming growth factor-β [11]. Insulin stimulates the countenance of the angiotensin II type 1 receptor in mesangial cells, magnifying the detrimental outcomes of angiotensin II in the kidney and enhancing the synthesis and nephritic activity of endothelin-1 [12]. Besides, reduced endothelial nitric oxide (NO) generation and considerably oxidative stress are allied to IR and hyperinsulinemia, which participate in the progression of DN [13]. This review explains the aforementioned and additional putative pathways through which IR and hyperinsulinemia may cause renal injury.

Materials and methods

**Figure 1:**
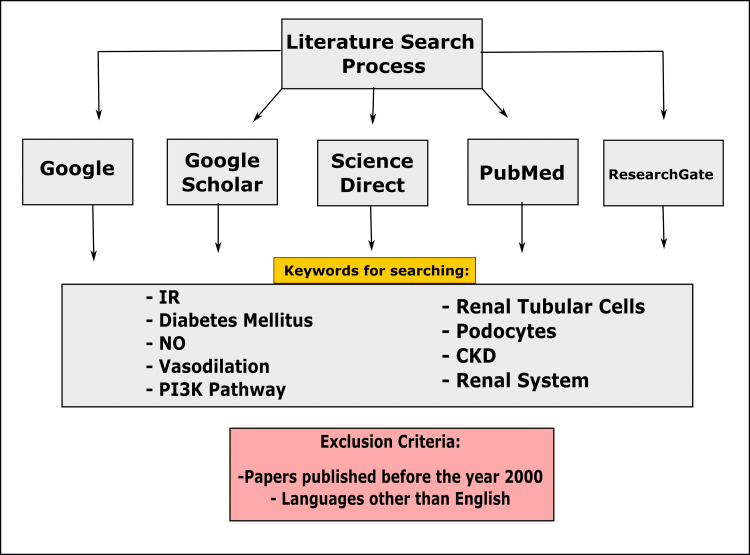
Diagrammatic representation of the study method. IR: insulin resistance; NO: nitric oxide; PI3K: phosphoinositide 3-kinase; CKD: chronic kidney disease Image credit: Susmita Sinha

This paper explains the probable means for insulin insensitivity, T2DM, and the renal system. The pieces of literature were searched on electronic archives through Google, Google Scholar, ScienceDirect, PubMed, and ResearchGate. The list of references of allied papers was reviewed to obtain additional pieces of literature. Keywords included IR, diabetes mellitus, NO, vasodilation, PI3K pathway, renal tubular cells, podocytes, CKD, and renal system. Papers published before 2000 and printed in other languages were cast aside (Figure 1). The appropriateness of the articles was manually checked before adding to this study. Identical articles were carefully removed. Following our valuation and assimilation of the suggested works of literature, a follow-up conversation was held to address any questions, concerns, errors, or biases related to the specific articles.

## Review

Podocytes and their delicacy

**Figure 2:**
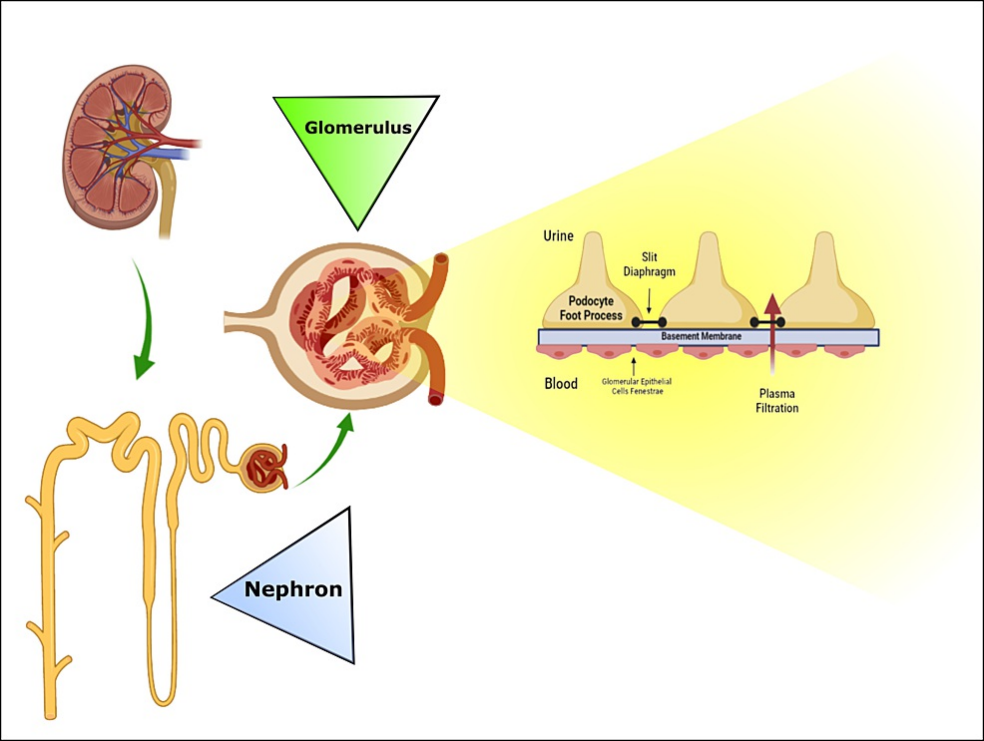
Schematic drawing of a typical nephron, glomerulus, and glomerular filtration barrier. Image credit: Susmita Sinha

Endothelial cells, the glomerular basement membrane, and podocytes construct the selective glomerular filtration barrier, which restricts protein loss from the blood into the dominant filtrate [14]. Slit diaphragms (SDs) are unique cell-to-cell connections formed by mature podocytes between interdigitating foot processes (Figure 2). The principal diameter-specific filtration system in the kidneys, for example, the SDs, is 20 nm in length and is essential for preserving glomerular structure and efficiency [15]. Podocytes have a compound actin filament cytoskeletal structure attributed to nephrin, the SD protein that serves as an organizational and signaling particle in the SD. Again, podocytes are incapable of proliferation; as a result, they are the most sensitive part of the glomerular filtration system. High glucose levels, increased free fatty acid echelons, free radicals, transforming growth factor-β, and angiotensin II, as well as hemodynamic influences such as structural stress caused by changes in capillary tension, all can cause podocyte damage [16].

Podocytes use glucose as energy

Podocytes’ primary power source is thought to be glucose, and these cells mainly acquire their energy through anaerobic glycolysis. Almost 80% of glucose is transported into podocytes by facilitative diffusion; the rest, 20%, presumably accounts for sodium-dependent cotransport [17]. Glucose transporter type 4 (GLUT-4) and 8 (GLUT-8) transport glucose into the cells through facilitated diffusion in podocytes. Moreover, sodium-glucose cotransporter 1 (SGLT-1) and 2 (SGLT-2) use the transmembrane sodium gradient for energy-dependent glucose uptake into the cells [18]. At standard conditions, GLUT-1 transports glucose into cells, while GLUT-4 transports glucose in the presence of insulin. GLUT-4 is typically confined to in-cell vesicular compartments initially; later, insulin stimulation causes a shift of GLUT-4 to the cell surface. In contrast to GLUT-1, GLUT-4 expression was depressed by prolonged exposure to podocytes to high glucose concentrations [19].

Effects of insulin on podocytes

**Figure 3:**
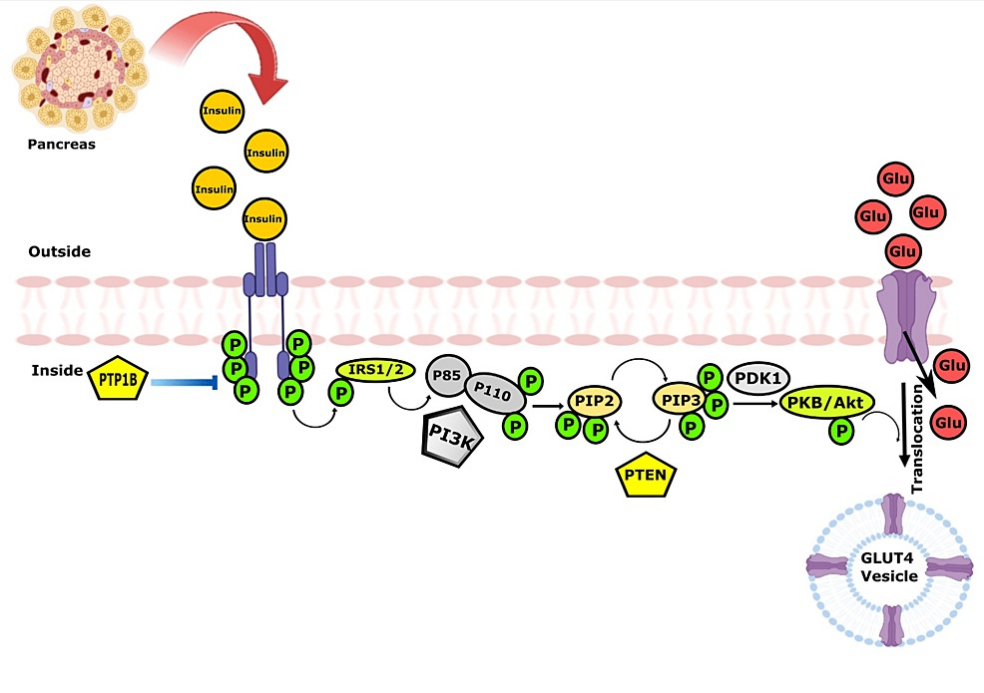
Insulin signaling pathway in podocytes. PTP1B: protein tyrosine phosphatase 1B; IRS: insulin receptor substrate; PI3K: phosphoinositide 3-kinase; PIP2: phosphatidylinositol 4,5-bisphosphate; PIP3: phosphatidylinositol 3,4,5-triphosphate; PTEN: phosphatase and tensin homolog; PDK1: phosphoinositide-dependent kinase-1; PKB/Akt: protein kinase B; GLUT-4: glucose transporter type 4 Image credit: Susmita Sinha

A membrane receptor protein is first bound to insulin, which then activates it to produce its subsequent outcomes. The insulin receptor is made up of four subunits grasped in conjunction by disulfide bonds: two α and two β subunits. The binding of insulin to the α subunits exterior to the cell membrane causes the cell to become autophosphorylated. This autophosphorylation of the β subunits of the receptor triggers tyrosine kinase, which in succession roots the phosphorylation of several other intracellular enzymes termed insulin receptor substrate (IRS) [20]. Diverse kinds of IRS are exhibited in various tissues. Phosphorylated IRS then binds to the controlling subunit of phosphoinositide 3-kinase (PI3K) for its activation. Activated PI3K phosphorylates phosphatidylinositol 4,5-bisphosphate (PIP2) to phosphatidylinositol 3,4,5-triphosphate (PIP3), subsequently rises in PIP3 accumulation at the plasma membrane, and later engages and stimulates phosphoinositide-dependent kinase-1 (PDK1). Protein kinase B (PKB/Akt) stimulation is the subsequent phase, needing its transportation to the cell surface, PIP3 attachment, and consequent phosphorylation by PDK1. Stimulated Akt provokes a shift of GLUT-4 from cytoplasmic sacs to the cell surface, ensuing in more C_6_H_12_O_6 _uptake into cells [21]. Thus, insulin directs the intracellular processes to provide the best impact on the metabolism of carbohydrates, fats, and proteins (Figure 3). In healthy people, insulin integrates the metabolism of carbohydrates, proteins, and lipids to maintain glucose homeostasis. Insulin limits fat and liver gluconeogenesis hydrolysis while enhancing glucose transport in muscle and the liver [22]. One more research study revealed that DN is also connected to higher morbidity and death rates in patients with diabetes as an important factor in renal failure. The physiopathological link involving T2DM and the renal system is triggered by a variety of extensive threat variables, notably heredity, overweight, hyperlipidemia, and resistance to insulin [23]. Furthermore, IR is the crucial linkage between these conditions.

Active insulin directly affects podocytes. Nephrin, one of the SD proteins, is vital in insulin-dependent C_6_H_12_O_6_ uptake into epithelial cells of renal glomerulus. In the presence of insulin, GLUT-4 vesicles in nephrin mutant podocytes were transferred to the cell’s boundary, but they were unable to attach to the cell surface [24]. Studies revealed that podocytes had more insulin receptors (IRs) than endothelium or mesangial cells, showing that insulin betokening in podocytes is all important for the activities of insulin [25]. Insulin links with the epithelial cells in the glomerulus to modify the actin cytoskeletal construction of the podocytes and is vital for preserving the functionality of the glomerular filtration barrier. Cytoplasmic tyrosine phosphatase Src homology 2 comprising protein tyrosine phosphatase (SHP-1) has been demonstrated to dephosphorylate a broad range of phosphor-proteins responsible for cell signaling of the tyrosine kinase family receptors. Reduced insulin and nephrin activities and glomerular epithelial cell malfunction were triggered by increased SHP-1 expression in podocytes exposed to hyperglycemia [26].

Type 2 diabetes mellitus and renal system

**Figure 4:**
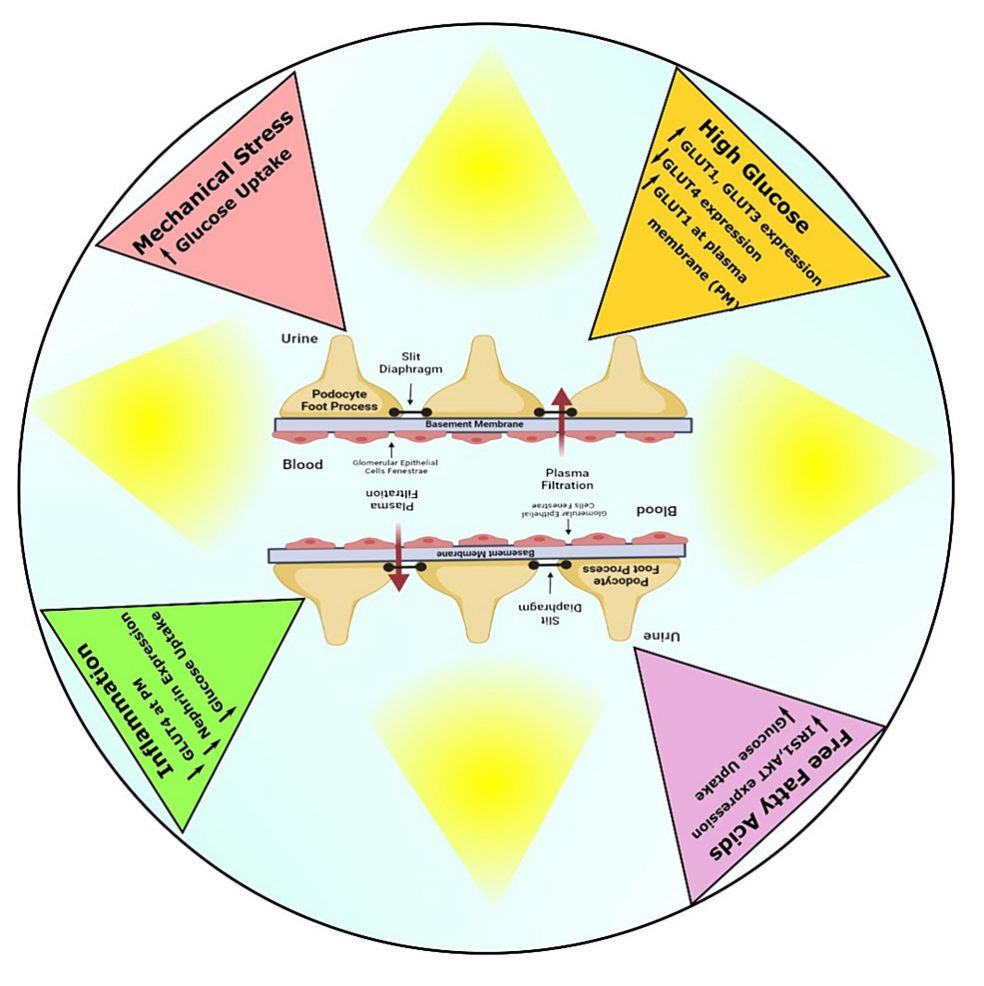
Schematic diagram showing the effects of diabetes on glucose transporters and glucose transport on podocytes. GLUT: glucose transporter; IRS1: insulin receptor substrate-1; Akt: protein kinase B Image credit: Susmita Sinha

Fasting plasma C_6_H_12_O_6_ echelons of over 126 mg/dL, oral glucose tolerance test (OGTT) values of over 200 mg/dL after two hours, HbA_1_C values of over 6.5%, or the usage of antidiabetic drugs are all indicators of diabetes mellitus [27]. Chronic hyperglycemia gradually leads to IR and is thereby associated with the pathogenicity of T2DM [28]. Rats fed a high-fat diet (HFD) along with the injection of low doses of streptozotocin (STZ) develop T2DM, which is an insulin-resistant condition [29]. Another study found that prostaglandin E1 (PGE1), one of the hormones most tissues produce to control blood flow, hindered IR and alleviated renal dysfunction in T2DM rats’ kidneys. Furthermore, they showed that PGE1-persuaded IR was restored as a consequence of the decrease in autophagy and subsequent overexpression of the resultant molecule fibroblast growth factor-21 (FGF-21) [30]. Numerous degenerative events in DN possibly are the major impairments in the stimulation by insulin and their transformation path in glomerular epithelial cells. Past research found a strong relationship between DN and glomerular podocyte structural damage and malfunction. In diabetic and nondiabetic glomerular pathologies, a decline in the proportion of podocytes results in proteinuria and glomerulosclerosis [31]. Exposition to elevated glucose concentration causes many cellular abnormalities in insulins’ usual target cells, such as muscles, adipocytes, and hepatocytes. Apart from the representative insulin target tissues, insulin affects the majority of human organs and cells, for instance, the kidneys and arteries, by modifying the hemodynamics, podocyte, and tubular function (Figure 4) [32].

Insulin resistance

IR is diminished physiological responsiveness to insulin instigation in target tissues, particularly the liver, muscle, and adipose tissue [33]. In addition, IR impedes glucose removal, giving rise to hyperinsulinemia, and IR is a widespread condition affecting numerous organs and insulin-regulated pathways [34]. Patients with mild to moderate CKD frequently have IR, individually identified as a nontraditional health concern and an important determinant of cardiac events in ESRD [35]. IR is frequent in ESRD subjects and accompanies malnourishment and protein energy depletion. Atypical insulin activity may lead to renal failure associated with nutritional, metabolic, and circulatory consequences. As a result, IR may represent a significant treatment option for lowering mortality in CKD patients. During IR, insulin augments the renal sodium reabsorption and provokes sympathetic nervous system work, which could significantly subsidize the blooming of hypertension [36].

Measurement of Insulin Resistance

The ideal technique for IR assessment is the hyperinsulinemic-euglycemic clamp because it offers a precise valuation of the overall physique’s sensitivity to insulin, particularly skeletal muscle. Using tagged glucose can help determine precisely how well insulin suppresses endogenous glucose production when administered at a lower dose. This technique affords a direct and detailed IR measurement and can differentiate between peripheral and hepatic IR [37]. IR can be evaluated by “Homeostasis Model Assessment of IR (HOMA-IR), calculated as insulin level in mIU/L times glucose in mg/dL divided by 405,” and “Matsuda index as a measure of whole-body IR, calculated as 10,000 divided by the square root of fasting plasma glucose (FPG) times fasting immunoreactive insulin (IRI) times two-hour post-load glucose times two-hour post-load IRI.” The Matsuda index measures resistance to insulin throughout the entire body, especially skeletal muscle, and is applied to quantify HOMA-IR, which indicates hepatic IR using the Homeostasis Model Assessments. In individuals with CKD, measurements of IR relying on fasting insulin levels may not be accurate since CKD inhibits insulin catabolism, and fasting insulin concentration primarily represents hepatic abnormalities [38].

Insulin Resistance in Chronic Kidney Disease

**Figure 5:**
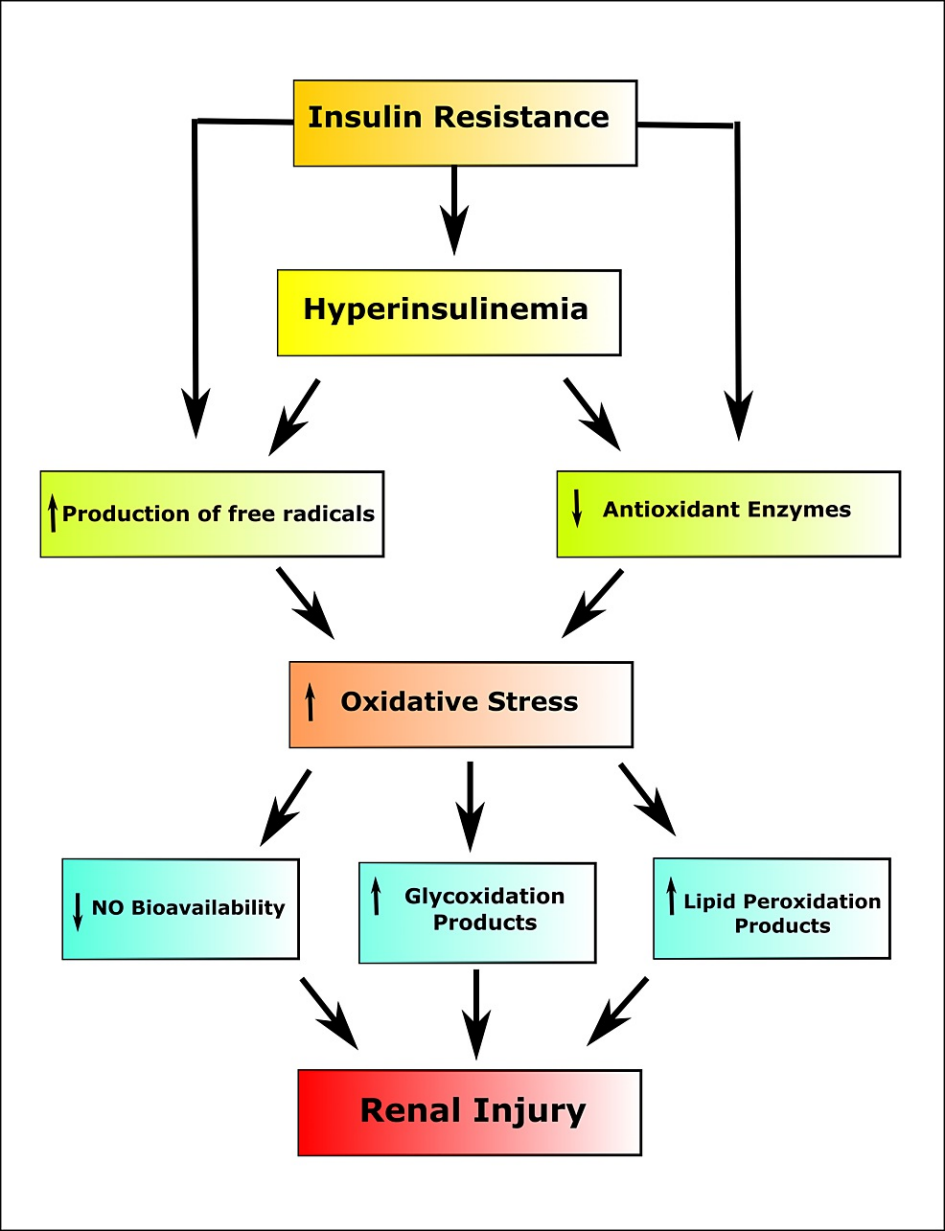
Process of the generation of renal injury in insulin resistance. NO: nitric oxide Image credit: Susmita Sinha

IR is carried on by impaired insulin signaling. Insulin combines with the insulin receptor on the cell surface of aimed tissues to generate the established biological responses [39]. IR is evident in the initial stage of CKD despite having an average glomerular filtration rate (GFR). It has been postulated that IR, along with oxidative stress and inflammation, performs a part in the headway of albuminuria and the decline of kidney function [40]. Hyperglycemia may induce poor renal function and reduced assertion of Klotho protein in the DM kidney. These abnormalities in diabetic rats could be reversed by lowering plasma C_6_H_12_O_6_ echelons with insulin therapy [41]. IR promotes kidney disease through deteriorating renal hemodynamics by processes including sympathetic nervous system stimulation, Na^+^ withholding, diminished Na^+^-K^+^ ATPase action, and higher GFR [42]. At the molecular level, endoplasmic reticulum (ER) stress appears to represent the link between the inflammatory process and IR [43]. A significant function is performed through the stimulation of c-Jun N-terminal kinase (JNK), which suppresses insulin summoning by phosphorylating the insulin receptor substrate-1 (IRS1) [44]. The underlying cause of proteinuria, kidney endoplasmic reticulum stress, is linked to podocyte injury resulting from proteinuria and changes in nephrin N-glycosylation in podocytes. Renal ER stress is also related to the pathogenesis of chronic kidney dysfunction with tubulointerstitial degeneration (Figure 5) [45]. The increased triglyceride levels in the blood and the over-production of very low-density lipoprotein cholesterol may be instigated by IR. Reactive oxygen species (ROS), which prevent insulin from inducing tyrosine autophosphorylation of the insulin receptor, have been hypothesized to perform a role in the evolution of IR [46].

In contrast to IR, which first manifests in the liver and then in white adipose tissue, the skeletal muscle remains liable to insulin [47]. The primary deficiency in CKD is accredited to be a post-receptor impairment [48]. CKD is influenced by a complex circuit of dietary and metabolic changes involving oxidative stress, IR, chronic inflammation, and protein energy wastage. Patients with CKD have low-grade inflammatory reactions similar to that seen in most chronic illnesses, as seen by higher echelons of pro-inflammatory cytokines, notably C-reactive protein (CRP), tumor necrosis factor-alpha (TNF-α), interleukin-6 (IL-6), and interleukin-1 beta (IL-1β) [49]. Inflammation and oxidative stress are known to cause IR, mostly by the higher production of pro-inflammatory cytokines, and are noticeable in the initial phases of CKD. The phosphoinositide 3-kinase (PI3K)-Akt/protein kinase B (PKB) pathway, which is in charge of the majority of metabolic processes such as glucose transport, and the Ras/mitogen-activated protein kinase (MAPK) pathway, which controls gene expression and works in conjunction with the PI3K pathways to regulate cell proliferation and distinction, are the two main beckoning pathways that are triggered in the presence of insulin [50].

TNF-α infusion causes IR in skeletal muscle [51], which is connected with decreased phosphorylation of Akt substrate 160, resulting in the impairment of GLUT-4 shift and C_6_H_12_O_6_ utilization [51]. Another research shows that IL-6, too, can hinder the insulin signaling pathways at the insulin receptor and IRS1 communication levels, and the progression of IR is due to errors in insulin signaling via the protein kinase Akt [52].

Insulin Signaling Derangements

The insulin-induced growth factor receptor-bound protein 2 (Grb2)-son of sevenless (SOS)-renin-angiotensin system (RAS) pathway activates mitogen-activated protein kinase (MAPK) extracellular signal‑regulated protein kinases (ERKs)-1/2. It is a crucial regulator of cell division and proliferation [53]. Insulin also has non-metabolic actions conducted through the PI3K/Akt/PKB pathway, such as preventing apoptosis and encouraging cell viability. To prevent severe metabolic and proliferating disturbances, insulin signal transduction should be closely regulated [54]. To block the signal pathway in the important insulin receptor/IRS or Akt/PKB, the negative controls are frequently stimulated by insulin as a feedback system. Chronic hyperactivation of these regulators results in their dysregulation, which leads to IR [55]. Phosphotyrosine and phosphoserine/threonine protein phosphatases (PTP1B and PP2A, B, and C) are examples of negative controllers, lipid phosphatases regulating PIP3 levels (PTEN and SHIP) [56,57] and insulin receptor adaptor proteins and IRS (Grb and SOCS). Serine/threonine phosphorylation of the insulin receptor and IRS by insulin-mediated activation of serine/threonine kinases, primarily by a c-Jun amino-terminal kinase (JNK), IkB kinase (IKK), protein kinase C (PKC), serine/threonine-protein kinase (S6K1), and ERKs, is an additional conventional inhibitory process of the insulin signaling cascade [58,59].

Effects of Insulin on NO Signaling and Its Contribution to Developing Hypertension

Various organs have shown hemodynamic consequences of insulin, suggesting that insulin has a widespread NO-mediated vasodilatory impact. Insulin stimulates PI3K, PDK1, and Akt/PKB after binding to endothelial IR, which increases endothelial nitric oxide synthase (eNOS) activity and promotes NO generation by phosphorylating eNOS at Ser1177 [60]. Additionally, insulin activates the MAPK/ERK pathway, which causes endothelial cells to generate and secrete endothelin-1, promoting vascular smooth muscle cell (VSMC) growth and vasoconstriction. The MAPK/ERK pathway is unaffected or strengthened in the IR condition when the PI3K route is compromised, resulting in a reduction in NO generation, since both courses are out of balance, leading to vasoconstriction, VSMC growth, and consequently hypertension [61,62].

Insulin Resistance and Renal Blood Flow

As shown by eliminating para-aminohippuric acid (PAH) throughout a hyperinsulinemic-euglycemic clamp, insulin enhanced renal blood flow in physiological circumstances. L-N-monomethyl-L-arginine, an antagonist of NO synthase, reversed this outcome [63], demonstrating that insulin encouraged NO production in the renal vasculature. It is assumed that the renal vasculature exhibits diminished NO signaling, indicative of an IR condition [64]. Renal blood flow would be diminished as a consequence of the increased renovascular resistance brought on by endothelial dysfunction of the renal arteries [65]. GFR would be decreased as a result of decreased insulin-stimulated NO generation, causing an increase in renal vascular resistance [66]. GFR is frequently higher in obese individuals with syndrome X or uncontrolled T2DM [67]. Glomerular hyperfiltration, which characterizes early DN and is also present in type 1 diabetes mellitus, is most likely produced by factors apart from insulin [68]. Reduced tubuloglomerular feedback and afferent arteriole dilatation brought on by more significant salt reabsorption, including glucose, are the main processes causing glomerular hyperfiltration [69].

## Conclusions

The kidney contributes an essential command in maintaining glycemic homeostasis. The generation and use of renal glucose are predominant aspects of glucose metabolism in humans, as shown by experiments using radiolabeled glucose. IR would occur from the multiplex adaptation of the manifestation of glucose in the blood brought on by kidney disease. People with CKD experience IR at abundant stages of renal damage. Additional experiments and clinical research are required to understand the effects of insulin among individuals with CKD because enough data is not easily obtainable for renal failure patients.

Being overweight, decreased physical activity, and unhealthy food patterns are a few common but changeable health issues in CKD that also aggravate IR. Various IR targeting therapies such as lifestyle interventions, dialysis procedures, cessation of smoking, and regular exercise could be advantageous for CKD patients.
